# Structural Design and Optimization of the Milling Force Measurement Tool System Embedded with Thin-Film Strain Sensors

**DOI:** 10.3390/mi14122133

**Published:** 2023-11-21

**Authors:** Xiangtao Song, Wenge Wu, Yongjuan Zhao, Yunping Cheng, Lijuan Liu

**Affiliations:** School of Mechanical Engineering, North University of China, Taiyuan 030051, China; sz202102010@st.nuc.edu.cn (X.S.); zyj@nuc.edu.cn (Y.Z.); ypchengbk@163.com (Y.C.); liulijuan@nuc.edu.cn (L.L.)

**Keywords:** thin-film strain sensor, cutting force, wet etching process, performance characterization

## Abstract

A milling force measurement tool system is designed with an elastic beam structure, which is divided into a two-end ring hoop compression sensor mode and a two-end square hoop compression sensor mode to improve the strain sensitivity. A simplified mechanical model of the elastic beam is established, and the relationship between the strain and force of the elastic beam under the action of three cutting force components is investigated, which can act a guide for subsequent milling force measurement tool system calibration tests. Thin-film strain sensors occupy a central position in the milling force measurement tool system, which consists of a substrate, transition layer, insulating layer and resistance grid layer. The resistance grid layer has a particularly significant effect on the thin-film strain sensor’s performance. In order to further improve the sensitivity of thin-film strain sensors, the shapes of the substrate, the transition layer, the insulating layer and the resistance grid layer are optimized and studied. A new thin-film strain sensor is designed with a resistance grid beam constructed from an insulating layer and a resistive grid layer double-end-supported on the transition layer. The flow of the wet-etching process of thin-film strain sensors is studied and samples are obtained. The surface microforms of the sensor samples are observed by extended depth-of-field microscopy, confocal microscopy and atomic force microscopy. It can be seen that the boundary of the resistance grid layer pattern is tidy and has high dimensional accuracy, thus enabling the basic achievement of the expected effect of the design. The electrical performance of the samples is tested on an experimental platform that we built, and the results show that the resistive sensitivity coefficient of the samples is increased by about 20%, to 51.2%, compared with that of the flat thin-film strain sensor, which fulfils the design’s requirements.

## 1. Introduction

With the gradual development of MEMS, the advantages of intelligence, integration and miniaturization are gradually becoming clear, and it thus provides a good method for the measurement of cutting force. So far, developing a means of assessing the milling force that arises in traditional cutting remains important and difficult. In order to study the effect of milling force on the milling process, the measurement of milling force is very important. In order to measure milling force accurately, many scholars have undertaken much work. Adolfsson and Stahl [1] developed a milling force sensor and proposed a new way to measure the milling force; with the measurement equipment they developed, it is possible to directly measure the cutting forces acting on each individual cutting edge. Andreas Albrecht et al. [2] measured the milling force by measuring the displacement of a rotating spindle, and this could compensate for the effect of interference during milling force measurement. G. Byrne and G.E. O ‘Donnell [3] monitored the cutting forces that arise in the online machining process with two ring piezoelectric sensors. Ihsan Korkut [4] designed a data acquisition system and connected it to a strain dynamometer to accurately measure cutting forces. In order to improve the accuracy of milling force measurement, the force-measuring element is studied. Süleyman Yaldız et al. [5] designed an octagonal ring milling dynamometer to measure cutting forces using strain gauges. Martin B. Jun et al. [6] used a ring force sensor to measure milling forces. G. Totis et al. [7] designed and manufactured a new type of rotary dynamometer for the online monitoring of the dynamic information related to cutting forces. Qiaokang Liang et al. [8] designed an E-type membrane force sensor and measured forces and moments on three axes. You Zhao et al. [9] measured cutting forces through two interleaved octagonal ring elastic elements. Shanshan Hu et al. [10] designed a floating beam-type six-axis elastomer, which offers guidance in the design of elastomer structure. In order to improve the sensitivity of the milling force-measuring element, strain sensors have also been studied. Xudong Cheng and Xiaochun Li [11] designed and fabricated a PdCr thin-film strain sensor, and embedded it in a metal structure to measure milling forces. Maximilian Mathis et al. [12] used a NiCr-C thin-film strain gauge to reduce the influence of strain deviation on sensor accuracy. Much research has been done on the preparation of thin-film strain sensors. Ruyuan Ma [13] studied the factors affecting the surface microstructure of TiN films, and explored the optimal process parameters. Vijay Petley et al. [14] studied the interfacial shear stress and stress distribution of NiCr films. Christine Taylor and Suresh K. Sitaraman [15] studied NiCr thin-film resistance grids of different sizes. Li Wang et al. [16] proposed a total suspension structure based on AlN. F. Samaeifar et al. [17] designed a platinum microhot plate with a suspended membrane structure. Madhu Santosh Ku Mutyala et al. [18] studied methods to improve the sensitivity of thin-film strain sensors in microcantilever beams. Muhammad Rizal et al. [19] proposed a four-claw structured force-measuring instrument, which achieved good strain, but showed room for optimization in terms of structural details and strain sensor performance.

A cutting tool system for measuring milling force based on thin-film strain sensors is proposed in this paper. Here, the resistive grid beam is supported on the double end of the thin-film strain sensor on the transition layer to increase strain and sensitivity.

## 2. Structural Design and Strain Principle of Milling Force Measurement Tool System

**Figure 1:**
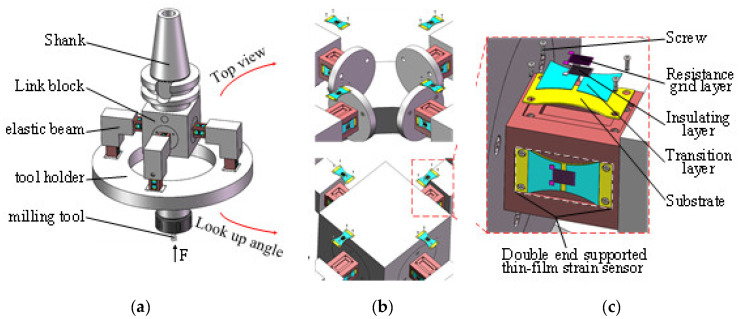
Milling force measurement tool system. (**a**) Tool system structure diagram; (**b**) elastic beam structure; (**c**) double-end-supported thin-film strain sensor.

As shown in Figure 1, the milling force measurement tool system with an elastic beam structure consists of a shank, a link block, four elastic beams, a tool holder, a milling tool, and a double-end-supported thin-film strain sensor. Each elastic beam consists of a horizontal beam and vertical beam. The thin-film strain sensors are mounted on both the horizontal beam and the vertical beam, transferring the strain signal generated by the milling forces to the resistance grid beam.

### 2.1. Structural Design

**Figure 2:**
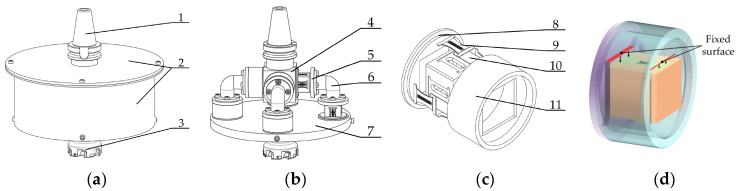
Milling force measurement tool system in the two-end ring hoop compression sensor mode. (**a**) Overall structure; (**b**) main structure; (**c**) two-end ring hoop compression sensor structure; (**d**) sensor in fixed mode. 1, shank; 2, protective round sleeve; 3, milling tool; 4, link block; 5, transfer disc; 6, connecting round beam; 7, tool holder; 8, annular hoop A; 9, double-end-supported thin-film strain sensor; 10, elastic square sleeve; 11, annular hoop B.

Figure 2 shows a milling force measurement tool system in the two-end ring hoop compression sensor mode. The system has twenty four double-end-supported thin-film strain sensors, and a protective round sleeve is installed on the outermost part. The deformation of the double-end-supported thin-film strain sensor caused by the milling forces causes a change in the strain value in the resistance grid layer of the sensor. Figure 2d shows the sensor fixed on a two-end ring hoop.

**Figure 3:**
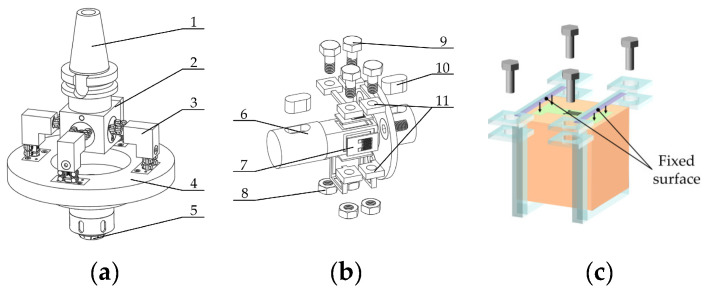
Milling force measurement tool system in the two-end square hoop compression sensor mode. (**a**) Overall structure; (**b**) two-end square hoop compression sensor structure; (**c**) diagram of sensor in fixed mode. 1, shank; 2, link block; 3, connecting square beam; 4, tool holder; 5, milling cutter chuck; 6, elastic horizontal beam; 7, double-end-supported thin-film strain sensor; 8, nut; 9, bolt; 10, key; 11, square hoop.

Figure 3 shows the milling force measurement tool system in two-end square hoop compression sensor mode. The structure of the tool system is similar to that shown in Figure 2, except that the sensor is fixed in a different way. As shown in Figure 3c, the sensor is fixed with square hoops at two ends.

**Figure 4:**
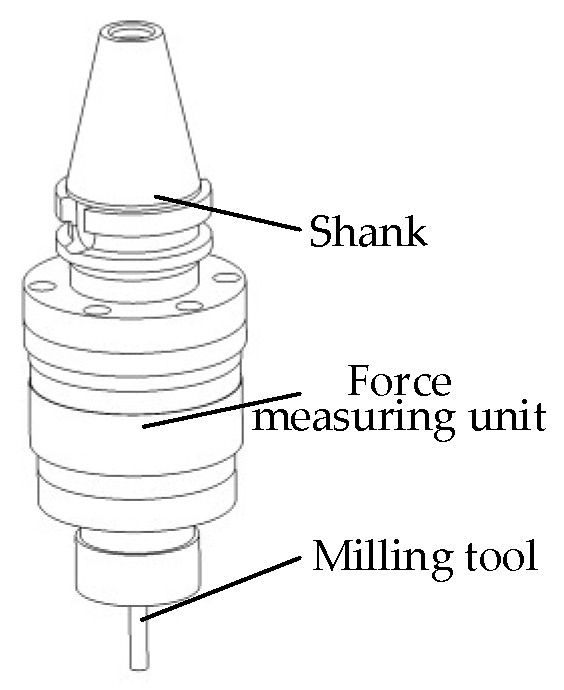
Milling force-measurement tool system for the cross-beam structure.

The milling force measurement tool system with a cross-beam structure consists of a shank, a force-measuring unit, and a milling cutter, as shown in Figure 4. The force-measuring unit has an internal cross-beam strain structure, similar to that shown in Figure 3.

### 2.2. Strain Principle of Milling Force Measurement Tool System

**Figure 5:**
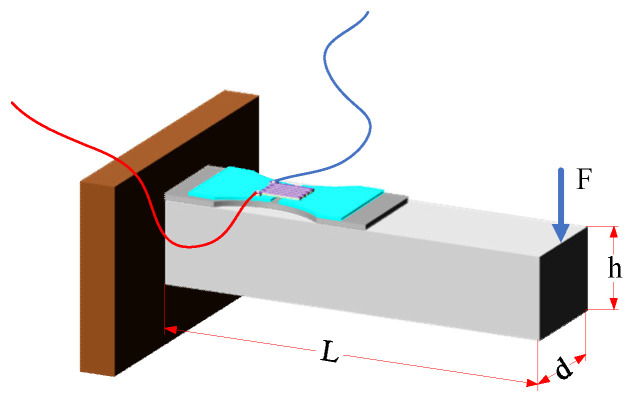
Schematic diagram of cantilever beam.

As shown in Figure 5, the elastic beam is identical in shape to the cantilever beam. When a force is applied to the free end of the cantilever beam, a change results in the strain value on the sensor. The stress–strain relationship of the cantilever beam can be defined as

(1) $$\varepsilon =\frac{\sigma }{E}=\frac{6FL}{Edh^{2}}$$

where *ε* is the strain, *σ* is the stress, *E* is the elastic modulus, *F* is the force on the free end of the cantilever beam, *L* is the moment length, *d* is the width of the cantilever beam, and *h* is the thickness of the cantilever beam.

**Figure 6:**
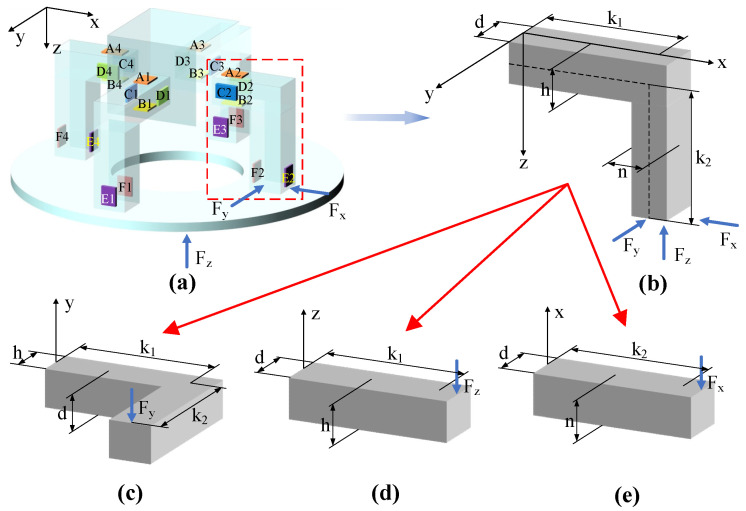
Elastomer model under three-directional cutting force. (**a**) Elastomer model and sensor distribution diagram; (**b**) schematic diagram of an elastic beam subjected to three-directional cutting force; (**c**) *y*-axis view; (**d**) *z*-axis view; (**e**) *x*-axis view.

The link block, four elastic beams and tool holder (Figure 1) constitute the elastomer model that is subjected to three-directional cutting force, as shown in Figure 6. As the installation positions of the elastic beam and the sensor are symmetrical, a simplified model can be established by selecting one of the four elements of the elastic beam.

The stress–strain relationship of one of the four elements of the elastic beam under the action of three-directional cutting force is here analyzed.

Moment equilibrium occurs on the neutral axis, so the arm of the moment gives the distance from the horizontal beam to the vertical beam, expressed as

(2) $$L_{k}=(k_{1}^{2}+k_{2}^{2}{)}^{\frac{1}{2}}$$

when the horizontal beam is subjected to a circumferential force, *F_y_*, as shown in Figure 6c. By substituting Equation (2) into Equation (1), the relationship between the circumferential force *F_y_* and the strain can be illustrated as

(3) $$\varepsilon_{F_{y}}=\frac{3F_{y}}{2Ehd^{2}}(k_{1}^{2}+k_{2}^{2}{)}^{\frac{1}{2}}$$

When the axial force *F_z_* acts on the bottom of the horizontal beam, as shown in Figure 6d, the arm of moment is *k*_1_, and the relationship between *F_z_* and the strain is

(4) $$\varepsilon_{F_{z}}=\frac{3F_{z}k_{1}}{2Edh^{2}}$$

The corresponding relationship between the radial force *F_x_* and the strain is

(5) $$\varepsilon_{F_{x}}=\frac{3F_{x}k_{2}}{2Edn^{2}}$$

The strain gauge factor is a fundamental parameter reflecting strain sensitivity, and can be expressed as [20]

(6) $$GF=\frac{\Delta R/R}{\Delta L/L}=\frac{\Delta R/R}{\varepsilon }$$

where *R* is the original resistance value of the resistance grid layer, *L* is the original length, and *ε* is the strain detected by the double-end-supported thin-film strain sensor.

**Figure 7:**
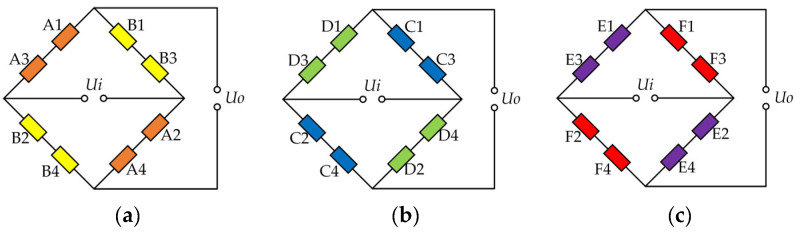
Wheatstone bridge connection diagram. (**a**) Wheatstone bridge for axial force *F_z_*; (**b**) Wheatstone bridge for circumferential force *F_y_*; (**c**) Wheatstone bridge for radial force *F_x_*.

The arrangement of thin-film strain sensors applied for detecting milling force is described in Figure 6a. The thin-film strain resistance sensors A1, A2, A3 and A4 are subjected to compressive stress, while the resistance sensors B1, B2, B3 and B4 are subjected to tensile stress, and these are all connected together to form a Wheatstone bridge circuit that can be used in detecting *F_z_*, as shown in Figure 7a. The circumferential force *F_y_* is detected by C1, C2, C3 and C4 when tensile stress occurs in these thin-film strain sensors, as shown in Figure 7b, while D1, D2, D3 and D4 are subjected to compressive stress. The radial force *F_x_* is applied to the four beams with thin-film strain resistance sensors, as shown in Figure 7c. Tensile stress occurs on the thin-film strain resistance sensors E1, E2, E3 and E4, while thin-film strain resistance sensors F1, F2, F3 and F4 are subjected to compressive stress. The following formula is used to obtain the output voltage

(7) $$\frac{U_{o}}{U_{i}}=GF\varepsilon$$

where *U_i_* is the input voltage, *U_o_* is the output voltage, *GF* is the strain gauge factor, and *ε* is the strain.

When the double-end-supported thin-film strain sensors are set up in a linear system, the relationship can be described as:

(8) $$U=\left[\varepsilon \right]F$$

(9) $$\left[\begin{array}{c} U_{F_{y}}\\ U_{F_{z}}\\ U_{F_{x}} \end{array}\right]=\left[\begin{array}{ccc} \varepsilon_{11} & \varepsilon_{12} & \varepsilon_{13}\\ \varepsilon_{21} & \varepsilon_{22} & \varepsilon_{23}\\ \varepsilon_{31} & \varepsilon_{32} & \varepsilon_{33} \end{array}\right]\left[\begin{array}{c} F_{y}\\ F_{z}\\ F_{x} \end{array}\right]$$

where *F* is the vector of the input component force value [*F_y_ F_z_ F_x_*], [*ε*] is the strain compliance matrix, and *U* is the vector of the output voltage value. Using the calibration platform, the output voltage generated by the double-end-supported thin-film strain sensor under three-directional cutting force loading can be measured, and when this process is combined with the above theoretical deduction, the milling force measurement tool system can be subjected to a calibration test.

## 3. Design and Preparation of Double-End-Supported Thin-Film Strain Sensor

### 3.1. Design of Double-End-Supported Thin-Film Strain Sensor

#### 3.1.1. Structural Design of Double-End-Supported Thin-Film Strain Sensor

The double-end-supported thin-film strain sensor is composed of a substrate and thin-film system. The substrate’s material and structure, and the thin-film system, are the main factors affecting the sensitivity of the thin-film strain sensor. The material of the elastic beam is 40Cr Steel (Shanghai Jiaoguan Industrial Co., Ltd., Shanghai, China), and the material of the substrate is stainless steel (Shanghai Baowei Industrial Co., Ltd., Shanghai, China).

**Figure 8:**
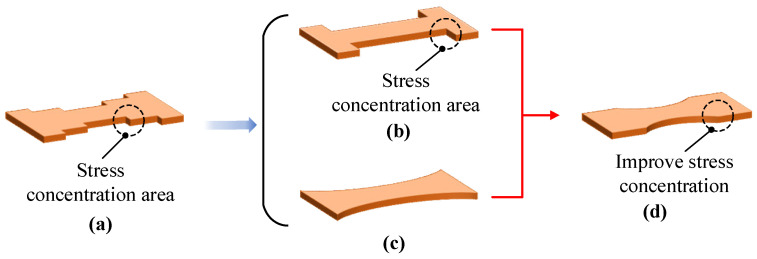
Optimization of the substrate. (**a**) Stepped-type substrate; (**b**) H-type substrate; (**c**) arc-type substrate; (**d**) arc transition H-type substrate.

A stepped-type substrate with good strain sensitivity has been designed, as shown in Figure 8a. But this structure has many right-angles, and stress concentrations occur at right-angles. The strain sensitivity values of the H-type substrate (Figure 8b) and the arc-type substrate (Figure 8c) are second only to that of the stepped-type substrate. The surface strain distribution of these two substrates was increased, but the degree of reduction in their stiffness was small. Therefore, combining these two substrates, an H-type substrate with a transitional arc has been designed, as shown in Figure 8d.

**Figure 9:**
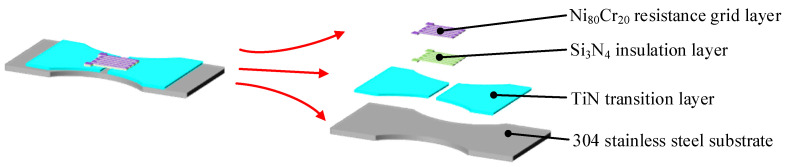
Double-end-supported thin-film strain sensor.

The thin-film system shown in Figure 9 is composed of a transition layer, an insulating layer and a resistance grid layer. Because the Si_3_N_4_ thin-film has a high dielectric constant and good density, it is suitable for use as an insulating material in microelectronics applications, so the material of the insulating layer is Si_3_N_4_. Previous studies have shown that the capacity for binding between the insulating layer and the substrate was not good, so a transition layer was added between the substrate and the insulating layer to ensure the insulating layer could grow well on the substrate’s surface. The film–substrate binding capacity, using a TiN thin-film and 304 stainless steel substrate, is better; the film-binding capacity is also good when using a TiN thin-film and a Si_3_N_4_ thin-film. Therefore, TiN was selected as the material of the transition layer thin-film. The material and structure of the resistance grid layer seriously affect the performance of the sensor. When using nichrome (Ni_80_Cr_20_) as the resistance grid layer, it can work normally at high temperatures below 450 °C.

**Figure 10:**
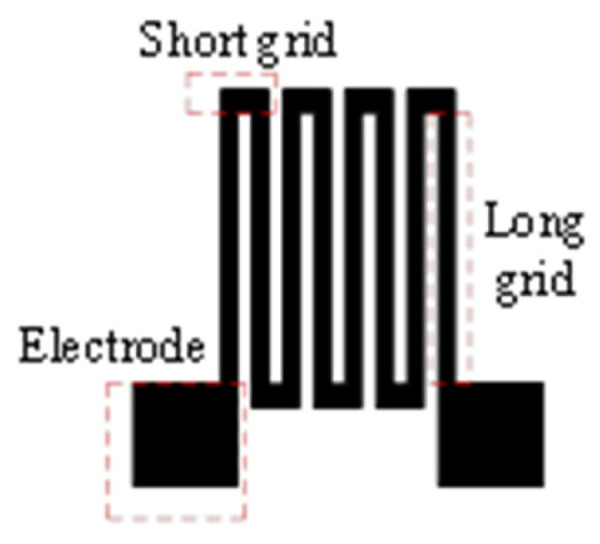
Resistance grid.

**Figure 11:**
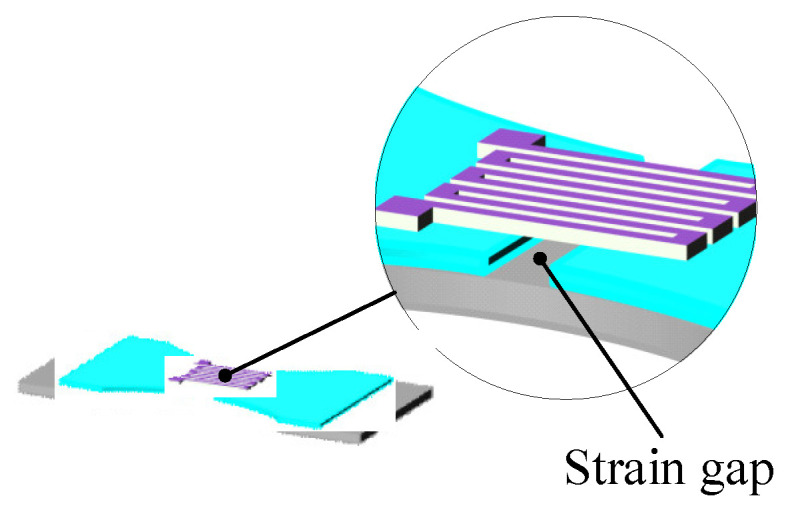
Double-end-supported structure.

In order to improve the sensitivity of the thin-film strain sensor, a double-end-supported thin-film strain sensor is designed with the structure of an ordinary thin-film strain sensor. As shown in Figure 9, a strain gap was established on the transition layer. The insulating layer and resistance grid layer have the same shape, while the two layers overlap to form a composite resistance grid. As shown in Figure 10, the resistance grid is composed of electrodes, long grids and short grids. As shown in Figure 11, the long grid of the composite resistance grid is arranged on the strain gap, and is perpendicular to the strain gap.

#### 3.1.2. Dimensional Design of Double-End-Supported Thin-Film Strain Sensor

The total length of the substrate of the double-end-supported thin-film strain sensor is 30 mm, and its total width is 16 mm. The sensing area is a rectangle of 16 mm × 8 mm, and the radius of the eight transition arcs is about 2 mm.

The long grid’s size is 2.1 mm × 0.05 mm, the short grid’s size is 0.2 mm × 0.1 mm, the thickness of the resistance grid layer is 1 μm, and the resistance grid comprises 10 long grids and nine short grids. The width of the strain gap is about 0.1 mm. The depth of the strain gap is about 500 nm.

### 3.2. Preparation Process of Double-End-Supported Thin-Film Strain Sensor

**Figure 12:**
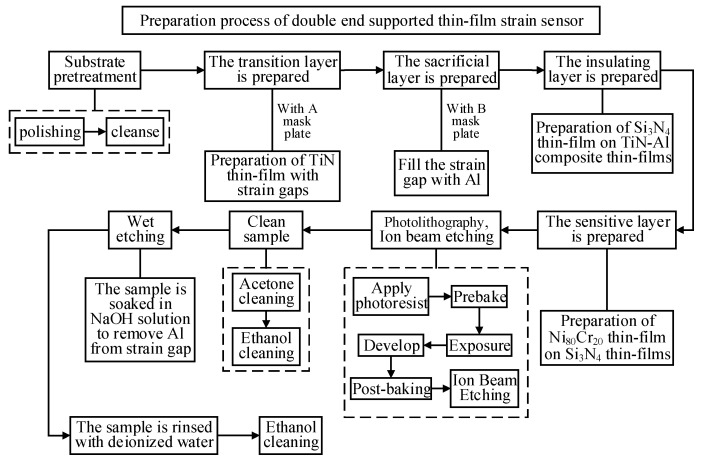
Flowchart of preparation process of double-end-supported thin-film strain sensor.

Figure 12 shows the preparation process of the double-end-supported thin-film strain sensor. The preparation process includes cleaning, magnetron sputtering deposition, photolithography, ion beam etching and wet etching. The pretreatment of the substrate, the removal of the photoresistor after ion beam etching and the removal of residual etching liquid after wet etching are all cleaning processes. The preparation of the transition layer, the sacrificial layer, the insulating layer and the sensitive layer comprise the magnetron sputtering coating process. Finally, the double-end-supported thin-film strain sensor is completed by cleaning it.

**Figure 13:**
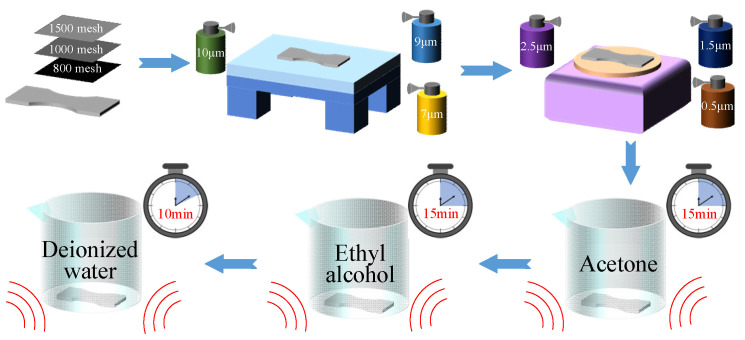
Pretreatment of substrate.

Figure 13 shows the pretreatment process applied to the substrate. Firstly, the substrate needs to be polished. See Table 1 for the detailed description of these steps.

After finishing polishing, the surface roughness Ra of the substrate will be less than 0.2 μm, thus meeting the requirements of the thin-film. Next, the substrate is cleaned to remove fine particles and any oil film that might remain on the surface of the substrate. The detailed steps of this procedure are shown in Table 2.

**Figure 14:**
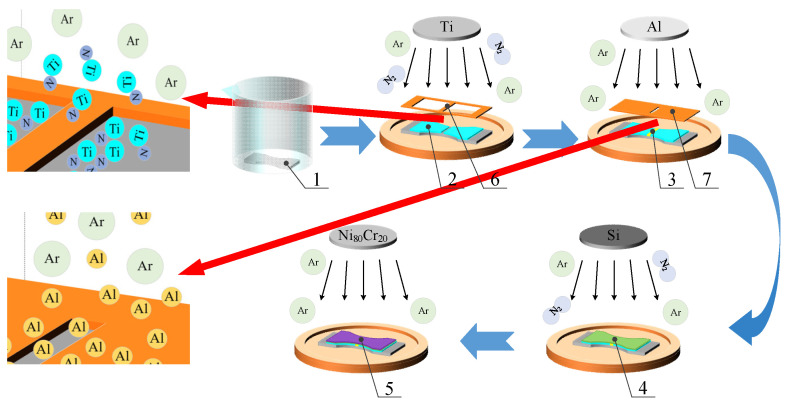
Magnetron sputtering deposition process. 1, substrate; 2, TiN thin-film; 3, Al sacrificial layer; 4, Si_3_N_4_ thin-film; 5, Ni_80_Cr_20_ thin-film; 6, A-mask plate; 7, B-mask plate.

Figure 14 shows the magnetron sputtering deposition process. First, the transition layer was prepared, and the TiN thin-film with a strain gap was prepared by magnetron sputtering, using as a base the A-mask plate. The thickness of the TiN thin-film was about 500 nm. Due to the presence of the A-mask plate, blocking some of the TiN thin-film from being generated, a strain gap was formed, and the depth of the strain gap was about 500 nm. Next, the sacrificial layer is prepared. The A-mask plate of the previous step was removed and the B-mask plate was installed. The Al sacrificial layer was applied to fill in the strain gap using magnetron sputtering technology, and the thickness of this sacrificial layer was about 500 nm. Since the size of the gap of the B-mask plate corresponded to that of the strain gap, Al was deposited in the strain gap via the gap in the B-mask plate. Next, the insulating layer was prepared, the B-mask plate of the previous step was removed, and the Si_3_N_4_ thin-film was constructed on top of the TiN-Al composite thin-film by magnetron sputtering. The thickness of the Si_3_N_4_ thin-film was about 500 nm. Finally, the sensitive layer was prepared, and the Ni_80_Cr_20_ thin-film was generated on top ofthe Si_3_N_4_ thin-film by magnetron sputtering. The thickness of the Ni_80_Cr_20_ thin-film was 1000 nm ± 50 nm. Table 3 shows the parameters of the sputtering process.

**Figure 15:**
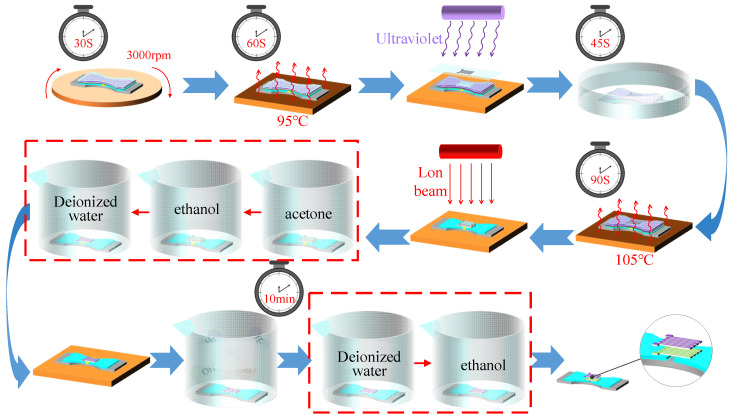
Photolithography, ion beam etching and wet etching processes.

As depicted in Figure 15, the photolithography process was carried out. An AZ4620 photoresistor was applied on the surface of the sample and homogenized using the homogenizing machine. After homogenizing, the thickness of the photoresistor on the surface of the sample was about 1.2 μm. The sample was prebaked and then exposed. The sample was immersed in the developer solution and studied with a microscope after development. After development had proceeded normally, the sample was post-baked and the photolithography process thus ended. Next, the sample was etched with an ion beam. The Ni_80_Cr_20_ thin-film and Si_3_N_4_ thin-film were etched onto the Ni_80_Cr_20_–Si_3_N_4_ composite resistance grid. After etching, the sample was cleaned to remove the residual photoresistor remaining on the surface. The details of the cleaning process are shown in Table 2. Finally, the wet etching process was carried out, and the Al in the strain gap was removed with a NaOH solution. After etching, the sample was put into the ultrasonic cleaning machine, and then cleaned with deionized water and ethanol. After drying, a double-end-supported thin-film strain sensor was obtained. Table 4 shows the parameters of the above process in detail.

## 4. Characterization of Microstructure and Electrical Properties of Double-End-Supported Thin-Film Strain Sensor

### 4.1. Characterization of Microstructure of Double-End-Supported Thin-Film Strain Sensor

**Figure 16:**
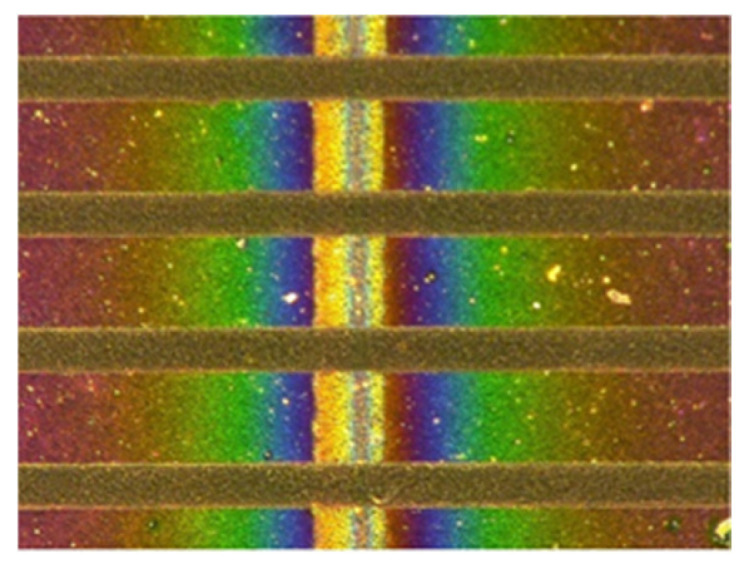
Microscopic morphology of double-end-supported thin-film strain sensor.

**Figure 17:**
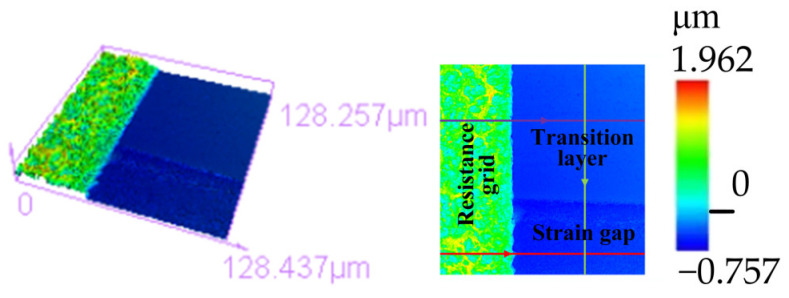
Height diagram of double-end-supported thin-film strain sensor.

As shown in Figure 16, the resistance grid was observed using extended depth-of-field microscopy. The boundary of the resistance grid and the etching pattern were determined with high accuracy. The resistance grid, transition layer and strain gap on the double-end-supported thin-film strain sensor’s surface could be clearly seen. As shown in Figure 17, a 128.437 μm × 128.257 μm cavity region consisting of a resistance grid, a transition layer and a strain gap was observed using the Confocal Microscope. The maximum height of the thin-film system was 1.962 μm, indicating that the distance from the bottom of the strain gap to the surface of the resistance grid was about 2 μm, which is basically consistent with the thickness of the designed thin-film system.

**Figure 18:**
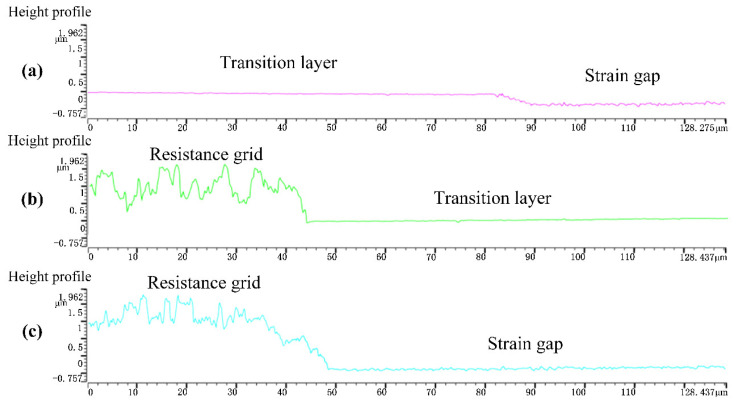
Height waveform diagram. (**a**) Transition layer to strain gap; (**b**) resistance grid to transition layer; (**c**) resistance grid to strain gap.

In Figure 18, the scanned regions between the transition layer and the strain gap (Figure 18a), the resistance grid and the transition layer (Figure 18b), and the resistance grid and the strain gap (Figure 18c) were all determined, and three sets of height waveform diagrams were obtained.

Figure 18a shows the height waveform diagram of the transition layer to the bottom of the strain gap, which was scanned in the direction shown by the green line in Figure 17. The height of the transition layer changed smoothly, indicating that the surface of the transition layer was relatively flat and the surface quality of the thin-film was good. When the scan reached about 82 μm, the resulting height waveform diagram began to declined, with a decline of about 450 nm, indicating that the depth of the strain gap was about 450 nm. At the same time, the height waveform diagram of the strain gap shows a small range with a sawtooth structure, indicating that a small amount of Al remained in the strain gap. The duration of wet etching can be extended to further reduce the residual Al without damaging the double-end-supported structure. Figure 18b shows a height waveform diagram between the resistance grid and the transition layer (in the direction shown by the purple line in Figure 17). The height waveform diagram of the resistance grid reflects a wide range, from 0.46 μm to 1.962 μm, with a roughly 1.5 μm difference in altitude between the resistance grid and the transition layer, indicating that when the surface roughness of the resistance grid is larger, the surface quality of the resistance grid will be affected. Photolithography and ion beam etching also affect the roughness of the resistance grid. Figure 18c shows that there is about 1.95 μm of altitude difference between the resistance grid and the bottom of the strain gap (along the direction shown by the red line in Figure 17).

**Figure 19:**
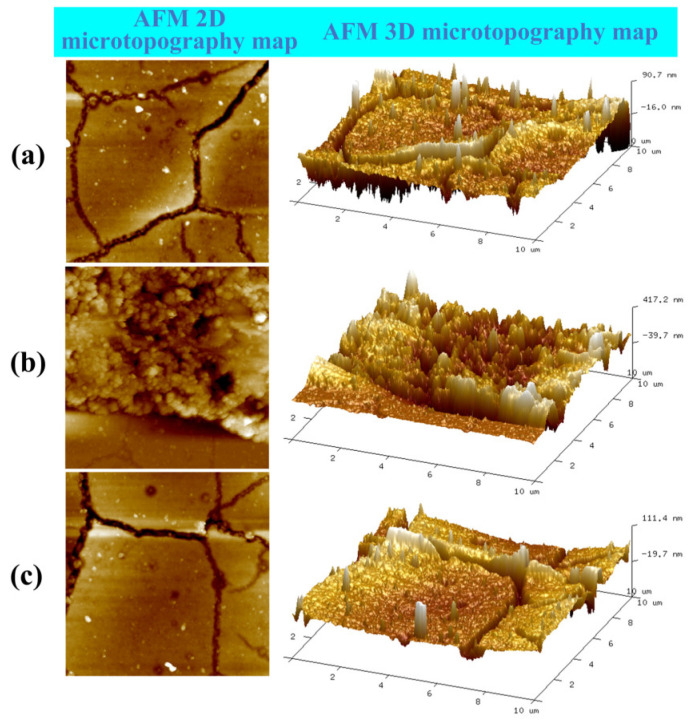
Micromorphology observation derived using AFM. (**a**) Resistance gate; (**b**) strain gap; (**c**) transition layer.

Figure 19 shows the results of microscopic morphology performed using an Atomic Force Microscope. The two-dimensional diagrams in Figure 19a,c show that cracks of different degrees appeared on the resistance grid and transition layer. It is thus inferred that the thermal stress generated in the process of prebaking and post-baking may cause cracks to emerge on the surface of the thin-film. A comparison of the three-dimensional diagrams shown in Figure 19a,c indicates that the flatness of the resistance grid surface is worse than that of the transition layer. There are very densely positioned height differences shown in the three-dimensional diagram in Figure 19b, and the microscopic morphologies of the surfaces with different heights are different. When these findings are combined with the above analysis of the height waveform diagram, it can be inferred that the residual Al remaining in the strain gap creates a region with very dense height differences, as shown in the three-dimensional diagram. In Figure 19b, the relatively smooth ribbon-shaped area is the stainless-steel substrate.

### 4.2. Electrical Performance Test of Double-End-Supported Thin-Film Strain Sensor

**Figure 20:**
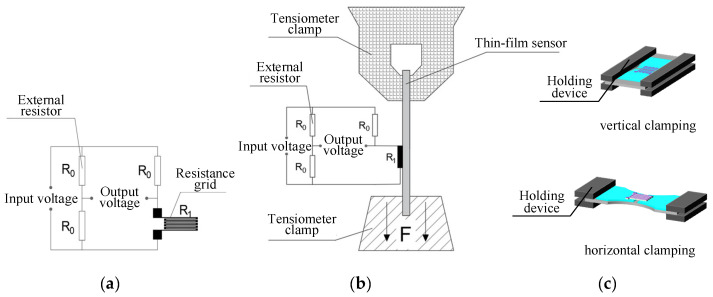
Electrical performance test. (**a**) Wheatstone bridge connection diagram; (**b**) test platform schematic diagram; (**c**) clamping mode.

**Figure 21:**
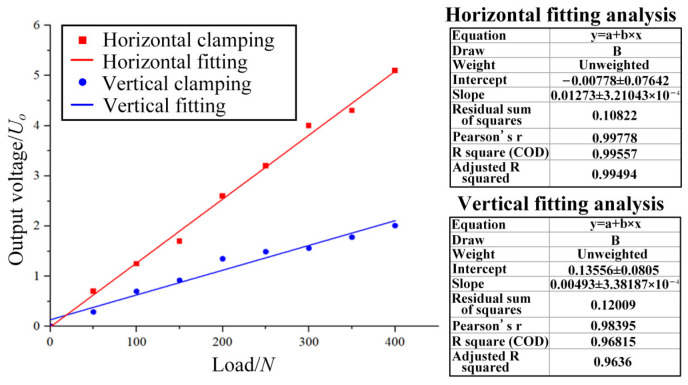
Relation between load and output voltage of double-end-supported thin-film strain sensor.

**Figure 22:**
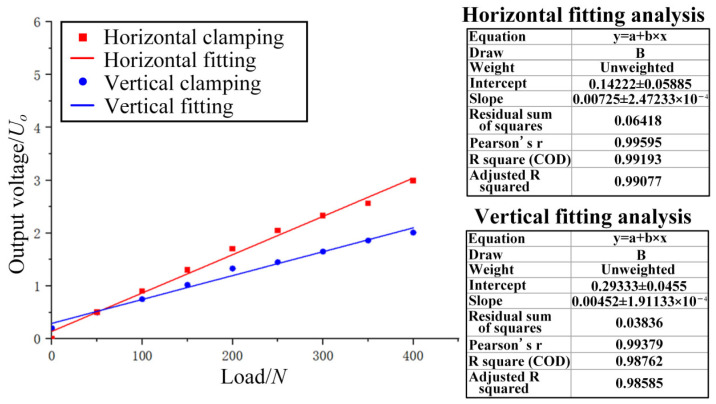
Relation between load and output voltage of ordinary thin-film strain sensor.

As shown in Figure 20, a test platform was built to determine the electrical performances of the ordinary thin-film strain sensor and the double-end-supported thin-film strain sensor, individually. Figure 20a shows a diagram of the Wheatstone bridge connection, wherein a resistance grid and three external resistors with the same resistance value are connected to form a quarter Wheatstone single bridge circuit; the input voltage here was 10 V, and the tension of 0–400 N was applied. As shown in Figure 20c, the two sensors are clamped horizontally and vertically, respectively. Eight groups of data were measured and subjected to fitting analysis, and finally, Figure 21 and Figure 22 have been obtained.

The fitting analysis in Figure 21 shows that the fitting value R^2^ is close to 1, indicating that the linear error of the fitting result is small, and the double-end-supported thin-film strain sensor has good linearity. Under the same load, the voltage generated using horizontal clamping is larger than that generated using vertical clamping, meaning horizontal clamping produces greater strain.

As shown in Figure 22, the ordinary thin-film strain sensor achieved good linearity, but the slope of the linear fitting curve produced when it was subjected to horizontal clamping is significantly less notable than that of the double-end-supported thin-film strain sensor. By comparing the slopes of the linear fitting curves of the two kinds of sensors, we can see that the sensitivity of the double-end-supported thin-film strain sensor was about 51.2% greater compared to the ordinary thin-film strain sensor.

According to Figure 21 and Equation (7), it can be concluded that the GF value of the double-end-supported thin-film strain sensor was approximately 2.46. According to Figure 22 and Equation (7), it can be concluded that the GF value of the ordinary thin-film strain sensor was approximately 1.73. As regards the gauge factor (GF), the sensitivity of the double-end-supported thin-film strain sensor was about 42.2% greater than that of the ordinary thin-film strain sensor.

### 4.3. Experimental Results

**Figure 23:**
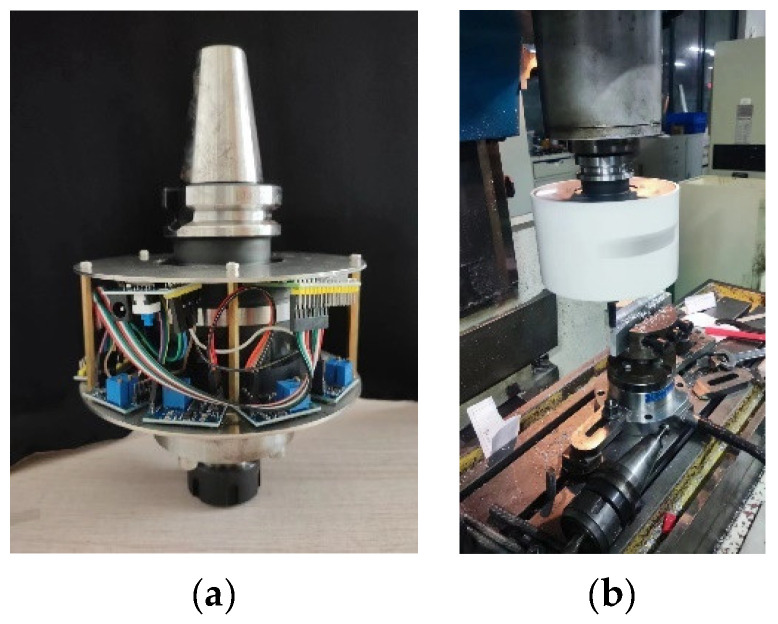
Cutting experiment applied to the milling force measurement tool system with a cross-beam structure. (**a**) Milling force measurement tool system with a cross-beam structure; (**b**) cutting test site diagram.

A comparative experimental study of cutting was conducted between the milling force measurement tool system with a cross-beam structure (Figure 4) and the Kistler dynamometer, under the same experimental conditions, as shown in Figure 23.

Experiment conditions: XK713 CNC milling machine (Wuhan Fourth Machine Tool Factory, Wuhan, China). Tools: *φ*10 mm carbide end mills. Workpiece material: 1060 aluminum alloy. The milling state was dry cutting, with a radial depth of the cut (*a_e_*) of 0.2 mm, an axial depth of the cut (*a_p_*) of 20 mm, a spindle speed of 1080 rpm and a feed speed of 80 mm/min.

**Figure 24:**
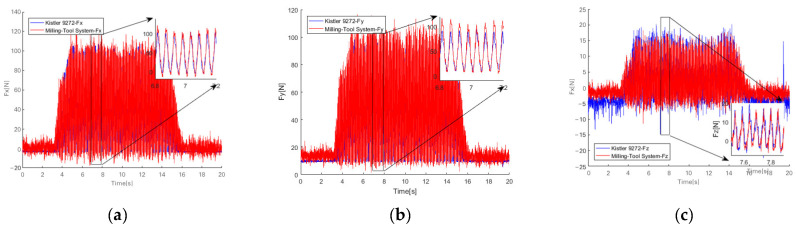
Plots of three-directional cutting force signals over time yielded by the milling force measurement tool system and the Kistler dynamometer. (**a**) Radial force F_x_; (**b**) circumferential force F_y_; (**c**) axial force F_z_.

In Figure 24, plots of the three-directional cutting force signal yielded over time by the milling force measurement tool system and the Kistler dynamometer are presented. From these, a table of the average values of the three-dimensional forces has been obtained, which can be used to compare between the force measuring system and the Kistler force measuring instrument, as shown in Table 5.

As illustrated in Table 5, the experimental results verify the validity of the milling force measurement tool system with a cross-beam structure.

## 5. Conclusions

1. In this paper, three kinds of double-end-supported thin-film strain sensors and milling cutter elastomers with an integrated structure are designed. The two milling force measurement tool systems used different sensor fixation methods;
2. We designed a double-end-supported thin-film strain sensor and refined its preparation process. All parameters of the preparation process, including cleaning, magnetron sputtering deposition, photolithography, ion beam etching process and wet etching, have been determined. Finally, a double-end-supported thin-film strain sensor was prepared;
3. By means of extended depth-of-field microscopy, confocal microscopy and atomic force microscopy, the double-end-supported thin-film strain sensors were studied. The results show that the resistance grid’s pattern boundary is regular, indicating higher etching accuracy. The transition layer and strain gap on the sensor’s surface have been clearly illustrated. The double-end-supported thin-film strain sensor met the design requirements;
4. The electrical performance of the double-end-supported thin-film strain sensor was tested. The test results show that the sensitivity of the double-end-supported thin-film strain sensor was about 51.2% greater than that of the ordinary thin-film strain sensor. Regarding the gauge factor (GF), the sensitivity of the double-end-supported thin-film strain sensor was about 42.2% greater compared to those of the ordinary thin-film strain sensors;
5. We conducted cutting tests on the milling force measurement tool system with a cross-beam structure. This system showed deviations of 13.7% in average radial force Fx, 7.12% in average circumferential force Fy and 10.33% in average axial force Fz compared to the Kistler dynamometer. The experimental results verify the validity of this system.

## Figures and Tables

**Table 1:** Polishing procedure.

	Equipment	Polishing Material		Treatment Method	Time
Step One		Sandpaper (Suzhou Suboli Grinding Materials Co., Ltd., Suzhou, China)	800 mesh 1000 mesh 1500 mesh	Hand Grinding	
Step Two	High-Strength Cast Iron HT200-300 Grinding Platform(Botou Bochuang Mechanical Equipment Manufacturing Co., Ltd., Cangzhou, China)	Diamond Spray Polish (Shenzhen Libaolixin Technology Co., Ltd., Shenzhen, China)	10 μm 9 μm 7 μm 5 μm 3 μm	Grind	30 min
Step Three	P-1 Type Metallographic Sample Polishing Machine (Shanghai Suoyan Testing Instrument Co., Ltd., Shanghai, China)	Diamond Spray Polish (Shenzhen Libaolixin Technology Co., Ltd., Shenzhen, China)	2.5 μm 1.5 μm 0.5 μm	Polishing	1 h

**Table 2:** Cleaning procedures.

	Equipment	Cleaning Solution	Time
Step One	Ultrasonic Cleaning Machine (Wenzhou Dongda Environmental Protection Equipment Co., Ltd., Wenzhou, China)	Acetone	15 min
Step Two		Anhydrous Ethanol	15 min
Step Three		Deionized Water	10 min

**Table 3:** Sputtering process parameters.

	Deposition Power	Pressure	Argon Flow Rate	Nitrogen Flow Rate	Time
TiN	100 W	1 Pa	50 sccm	3 sccm	380 s
Al	100 W	1 Pa	50 sccm		2.5 h
Si_3_N_4_	140 W	1.2 Pa	70 sccm	10 sccm	2 h
Ni_80_Cr_20_	100 W	1 Pa	70 sccm		1000 s

**Table 4:** Detailed process parameters.

Photolithography				
Technology	Equipment	Conditions		
**Apply Photoresistor (JSR Corporation)**	Cee200XCB (Beijing Saidekesi Electronics Co., Ltd., Beijing, China)	AZ4620 Photoresist	3000 r/min	30 s
Prebake	EH-20B Anti-corrosion Electric Heating Plate (Shenzhen Sanli Technology Co., Ltd., Shenzhen, China)		95 °C	60 s
Exposure	EVG 610 (Beijing Yake Chenxu Technology Co., Ltd., Beijing, China)	Film Paper Mask Plate	400 mJ/cm^2^	
Develop	Organic Hood (Shanxi Yitong Laboratory Equipment Co., Ltd., Taiyuan, China)	Developer Solution		45 s
Post-baking	EH-20B Anti-corrosion Electric Heating Plate (Shenzhen Sanli Technology Co., Ltd., Shenzhen, China)		105 °C	90 s
**Ion Beam Etching**				
**Equipment**	**Time**			
Ion Beam Etching Equipment (Beijing Midea Technology Co., Ltd., Beijing, China)	1 h 30 min			
**Wet etching**				
**Etching Solution**	**Time**			
3 mol/L NaOH Solution	10 min			

**Table 5:** Comparative analysis of the average values of the three-dimensional forces.

	F_x_	F_y_	F_z_
Kistler dynamometer	106.85	108.5	20.32
Milling force measurement tool system	121.49	116.22	18.22
Deviation value	13.70%	7.12%	10.33%

## Data Availability

The data used to support the findings of this study are available from the corresponding author upon request.
